# Advances in genetics, signaling, and modeling of venous malformations

**DOI:** 10.3389/fcvm.2026.1770126

**Published:** 2026-02-06

**Authors:** Komal Sagar, Elisa Boscolo

**Affiliations:** 1 Division of Experimental Hematology and Cancer Biology, Cincinnati Children’s Hospital Medical Center, Cincinnati, OH, United States; 2 Department of Pediatrics, University of Cincinnati College of Medicine, Cincinnati, OH, United States

**Keywords:** mouse model, PIK3CA, TIE2, vascular malformations, venous malformation

## Abstract

Vascular anomalies are defects resulting from the abnormal development or growth of the vasculature. Among these, venous malformations (VMs) are predominantly caused by mutations in the TIE2 or PIK3CA genes, which disrupt endothelial cell morphogenesis and vessel maturation. VM lesions are typically diagnosed during infancy or childhood and often persist and enlarge throughout adulthood, causing chronic complications such as pain, deformity, and coagulopathy. Despite available treatments such as sclerotherapy and mTOR inhibitors like sirolimus, achieving complete and long-term resolution of VMs remains a significant challenge. This review examines the genetic basis of VMs, explores the underlying molecular signaling mechanisms, and compares various experimental models—including *in vitro*, 3D, and *in vivo* systems—that have advanced our understanding of VM and provided platforms for testing potential therapies. Future research should prioritize the development of more precise and personalized models to drive improved strategies and better outcomes for patients with VMs.

## Introduction

Vascular malformations are diseases that can manifest with lesions in different types of vascular bed, such as veins, capillaries, lymphatics, and arteries. The 2025 Classification of Vascular Malformations by the International Society for the Study of Vascular Anomalies (ISSVA) (1) is based on slow or fast blood flow. Slow flow malformations include venous malformation (VM), capillary malformation (CM) and lymphatic malformation (LM), while fast flow malformations include arteriovenous malformation (AVM) and arteriovenous fistula (AVF).

Venous malformations are the most common type of vascular malformation, accounting for about 70% of cases (2, 3). VMs can be present at birth and enlarge progressively throughout life (4). Unlike vascular tumors, which are characterized by rampant endothelial cell proliferation, VMs stem from intrinsic defects in endothelial cell morphogenesis and signaling cascade. These defects impair proper vessel maturation and stabilization. Histologically, VMs are characterized by thin-walled ectatic veins with aberrant smooth muscle cell investment and disorganized extracellular matrix (ECM) (5, 6). Clinically, VMs manifest as soft, bluish, and compressible lesions. Affected patients experience symptoms such as chronic pain, severe disfigurement, swelling, localized thrombosis, and coagulopathy (5, 7). Genetic mutations resulting in the constitutive hyperactivation of the TIE2—PI3K signaling pathway are associated with VM. TIE2 is a receptor tyrosine kinase expressed primarily in endothelial cells. Its kinase domain activation by the ligand angiopoietin-1 (ANG-1) has a crucial role in blood vessel maturation and stabilization (8). Hyperactive mutant TIE2 can lead to increased PI3K (phosphoinositide 3-kinase) signaling leading to increased angiogenesis, endothelial cell growth, survival, and metabolism (9–11). It is not surprising that hyperactive mutations in the catalytic subunit of the PI3K (PIK3CA gene) have been identified in VM patients, leading to similar pathway activation and molecular consequences. Important studies highlighted in here demonstrate that these mutations drive excessive signaling while reducing vascular stability to promote the expansion of venous lesions (2, 10, 11).

In this review, we will focus on the groundbreaking advances in the genetics, molecular signaling characterization, preclinical experimental models, and therapeutic strategies in VMs (as summarized in Table 1). Combined, insights from cellular, *in-vitro* and *in-vivo* studies are set to fuel a better understanding of VMs.

**Table 1:** Timeline of venous malformation discoveries and experimental models.

Year	Major findings	References
1982	Classification and cell-oriented analysis of VMs	Mulliken et al. (109)
1996	TIE2 mutation identified in familial cases of VM	Vikkula et al. (34)
2008	Somatic non-inherited TIE2 mutations identified in VMs	Limaye et al, Wouters et al. (12, 14)
2015	Dysregulation of PI3K-AKT signaling as central for VM pathogenesis	Limaye et al, Uebelhoer et al, Natynki et al, Boscolo et al, (2, 12, 18, 40)
	PIK3CA mutation identified in subset of VM patients	Limaye et al. (20)
	Xenograft model VM with TIE2-L914F mutation	Boscolo et al. (40)
	Sirolimus identified as a potential therapy for VM	Boscolo et al. (40)
2016	Transgenic murine models with PIK3CA H1047R mutation	Castillo et al. (10)
		Castel et al. (11)
2016–18	Larger clinical trials identified Sirolimus as a potential therapy for VM	Adams et al. (82) Hammer et al. (83)
2018	Xenograft mice model with patient derived endothelial cell with TIE2 and PIK3CA mutations	Goines et al. (18)
2022	Use of 3D culture to study VM associated mutation	Boscolo et al. and Jauhainen et al, (93, 110)
2023	Alpelisib used in clinical trial for VM, found to reduce VM lesion size	Sterba et al. (16)
2023	Development of microfluidic device of VM by incorporating a physiologically relevant ECM	Aw et al. (17)
2024	Use of iPSCs to model VM	Lazovic et al, Pan et al, (98, 99)
2025	First genetic mouse model of TIE2-L914F	Bischoff et al. (105)

## Genetics of venous malformations

VMs are primarily driven by somatic, non-inherited mutations in the TIE2 and PIK3CA genes which disrupt endothelial cell signaling and vascular development. The most frequent genetic alteration in sporadic and rare familial form of VMs involves hyperactivating mutations in *TEK*, which encodes the endothelial receptor tyrosine kinase TIE2. These mutations are found in about 60% of VM cases (2, 3, 12, 13) and are typically localized in the intracellular kinase domains. Most of the VM causative mutations such as p.L914F, R849W, and Y897H are thought to disrupt the autoinhibitory loop at the C-terminal domain thereby leading to ligand-independent TIE2 phosphorylation and constitutive activation of the downstream PI3K–AKT pathway and, to a lesser degree, of the MAPK-ERK signaling (14). This hyperactivation promotes increased endothelial cell survival and impaired smooth muscle cell recruitment, likely leading to the formation of enlarged venous channels.

VM lesions in about 30% of patients harbor a mutation in *PIK3CA*, the gene encoding for the catalytic p110a subunit of PI3K (9, 11, 15–18). These genetic alterations often occur at activating residues such as E542K, E545K, or H1047R, which are common hotspot mutations in several types of cancer (19). Although PIK3CA and TIE2 mutations are mostly found to be mutually exclusive, a small number of cases with both mutations have been reported (18).

TIE2 and PIK3CA -mutated VM show distinctive anatomical and clinical features including lesion location and tissue depth that contribute to VM heterogeneity. TEK mutated-VM predominate in head and neck and trunk lesions, whereas PIK3CA mutations are largely confined to extremities with rare involvement of head and neck (15). In addition, lesion depth and tissue involvement also vary with the mutated gene. With TEK mutations, lesions frequently involve the skin and subcutaneous tissues and may extend into deeper structures, whereas PIK3CA-associated VMs present as deeper lesions with no skin involvement (20). Together, these findings indicate that different genetic drivers of VMs are associated with VM lesion variations in location, depth and phenotype.

Additional mutations in AKT1, RASA1, MAP3K3, and KRAS have been reported in atypical or mixed venous malformations (21–26). Crosstalk among these pathways indicates the complexity of vascular development and the shared signaling mechanisms across vascular anomaly subtypes.

While venous malformations (VMs) are predominantly driven by somatic mutations in the TIE2 and PIK3CA genes, other vascular malformations are associated with distinct molecular drivers, reflecting their unique vascular phenotypes (2, 3, 9, 11–13, 15–18). For instance, arteriovenous malformations (AVMs) frequently harbor activating mutations in KRAS, MAP2K1, or BRAF, which dysregulate RAS-MAPK signaling and promote high-flow vascular shunting (25, 27, 28). Capillary malformations (CMs) are often linked to GNAQ or GNA11 mutations, leading to altered G*α*q-mediated signaling and increased MAPK activation in endothelial cells (29–31). These contrasts highlight that while VMs primarily involve PI3K-AKT hyperactivation, AVMs and CMs arise through divergent signaling pathways, accounting for differences in flow dynamics, vessel morphology, and clinical presentation.

## Signaling in venous malformation

Two central molecular axes have been implicated in the VM pathogenesis: the TIE2 and PI3K-AKT pathway which share downstream effectors crucial for the maintenance of the blood vessel architecture.

**Figure 1:**
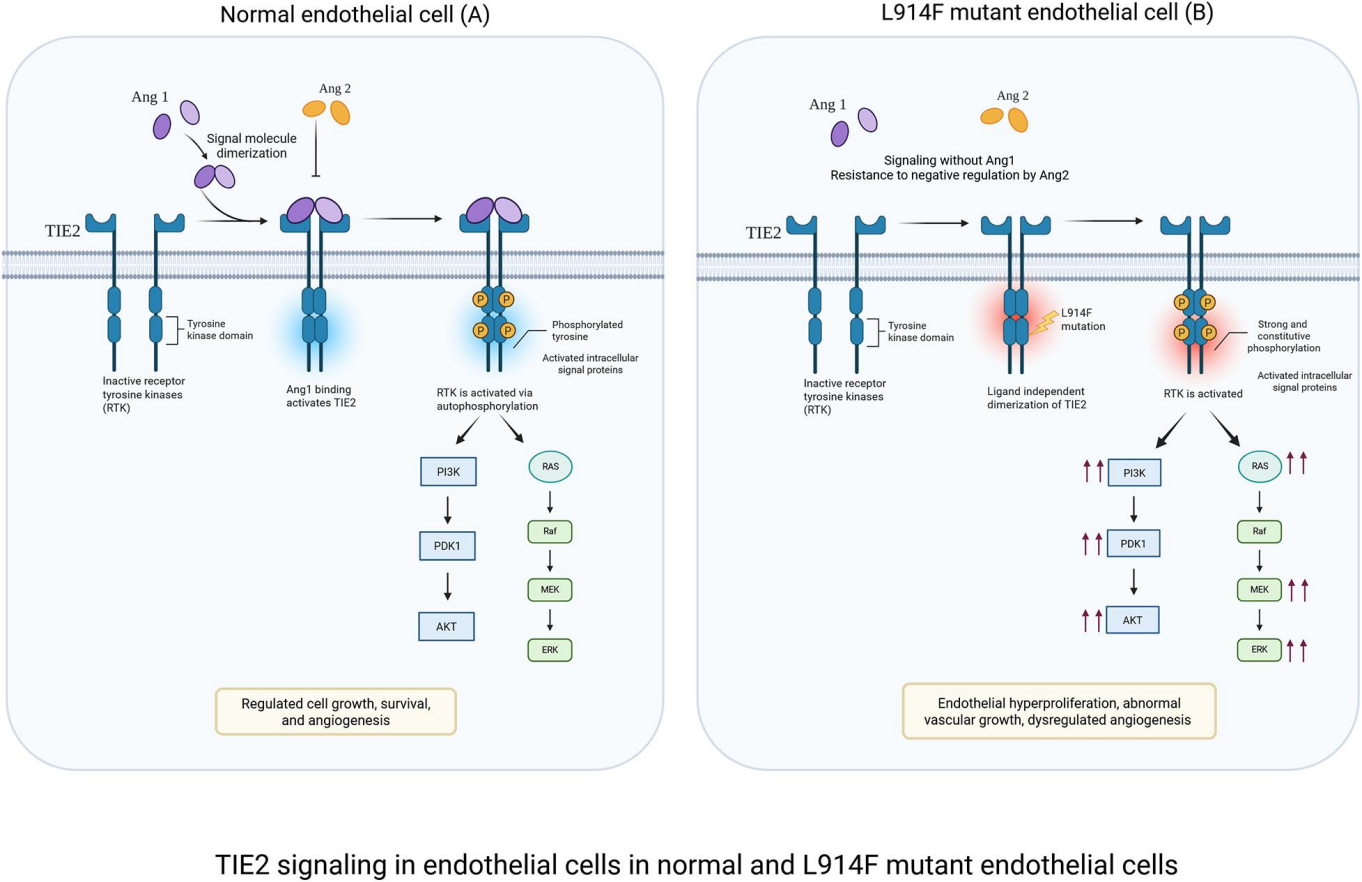
TIE2 signaling in endothelial cells under physiological and L914F-mutant conditions. **(A)** Physiological signaling: ANG-1 binding induces controlled TIE2 activation in normal EC, leading to balanced PI3K/PKT and MAPK/ERK signaling. This maintains endothelial quiescence, junctional stability, and vascular integrity. **(B)** L914F-mutant signaling: the L914F mutation causes ligand-independent TIE2 hyperactivation. Excessive PI3K/AKT and MAPK/ERK signaling results in increased endothelial cell survival, and the development of dilated vessels characteristic of venous malformations. Created in BioRender. Sagar, K. (2026), licensed under Academic License.

TIE2 receptor signaling: The receptor tyrosine kinase TIE2 is a bifunctional regulator of endothelial cell homeostasis or pathological angiogenesis (Figure 1) (2, 32). Under physiological conditions, TIE2 is modulated by the ligands angiopoietin-1 (ANG-1) and angiopoietin2 (ANG-2) (8). ANG-1 is a strong agonist for TIE2 by inducing receptor dimerization and tyrosine phosphorylation. This signaling promotes endothelial cell survival and vascular maturation by formation of mature cellular junctions and recruitment of perivascular cells such as pericytes and smooth muscle cells (8). In contrast, ANG-2 works as an antagonist of ANG-1, allowing vessels to remain plastic and responsive to VEGF- mediated angiogenesis (33). In VM, TIE2 activating mutations identified in VM patients lead to ligand-independent receptor activation and promote the constitutive activation of the PI3K-AKT signaling (18, 34). This persistent signaling not only amplifies downstream signaling but could disrupt the negative feedback regulation by ANG-2 (35). Consequently, vascular defects could occur, including disruption of endothelial barrier integrity, irregular endothelial cell morphology and excessive sprouting (15). Furthermore, mutant TIE2 signaling can indirectly influence the perivascular niche by altering the secretion of growth factors or extracellular matrix proteins implicated in mural cell recruitment, such as platelet-derived growth factor B (PDGF-B) and fibronectin, thereby contributing to the persistence of dilated, non-contractile venous channels (8, 12, 14, 34).

PI3K/AKT signaling: PI3K-AKT is a central pathway regulating processes such as proliferation, survival and metabolism (36). Upon activation, PI3K converts PIP2 into PIP3 recruiting and activating AKT. AKT then phosphorylates multiple downstream targets, including FOXO1 which restrains endothelial overgrowth and promotes vascular homeostasis (37, 38). Hyperactivation of AKT in VM promotes FOXO1 phosphorylation which prevents nuclear translocation and suppresses its transcriptional activity leading to endothelial hyperproliferation, aberrant vessel morphogenesis, and defective perivascular support (39). Both TIE2 and PIK3CA mutations converge on constitutive PI3K–AKT activation resulting in similar phenotypes of endothelial hyperplasia and aberrant lumen formation. Endothelial cells carrying TIE2 mutations exhibit additional activation of the MAPK/ERK pathway and STAT1 (14), whereas these pathways remain largely inactive in cells harboring PIK3CA mutations. Furthermore, PIK3CA-mutant endothelial cells show a markedly stronger AKT response compared to TIE2-mutant cells (2, 12, 18, 40). While hyperactive PIK3CA mutations are sufficient to cause the VM phenotype, it is tempting to downplay the role of the MAPK/ERK pathway in the VM genesis. However, recent studies in mice expressing endothelial hyperactive PIK3CA mutations highlighted a feedforward circuit converging on the hyperactivation of the TIE2 receptor which could in turn implicate MAPK in the formation and expansion of VM (39).

Beyond promoting endothelial survival and hyperproliferation, TIE2- or PIK3CA-driven hyperactivation disrupts endothelial cell polarity, a key determinant of proper lumen formation. Studies have shown that these mutations interfere with the apico-basal polarity and front-rear migration polarity, leading to misoriented intracellular junctions, defective lumen initiation, and irregular vessel diameter (41–43). This polarity disruption likely contributes to the ectatic, saccular vascular channels characteristic of VM, linking molecular signaling perturbations directly to the architectural abnormalities observed in lesions.

While hyperactivation of the TIE2–PI3K–AKT pathway has traditionally been viewed as a cell-autonomous driver of endothelial growth and survival, emerging evidence indicates that it also reshapes endothelial–endothelial cell communication in venous malformations. Recent studies demonstrate that TIE2-mutant endothelial cells gain a competitive advantage by actively repelling neighboring wild-type endothelial cells rather than recruiting them. This behavior is mediated by increased expression of the chemorepellent ligands Semaphorin-3A and Semaphorin-3F, which signal through Neuropilin-1/2 to suppress wild-type endothelial cell migration, sprouting, and lumen integration. Inhibition of Semaphorin 3A/3F signaling restored mutant–wild-type endothelial cell interaction and normalized pathological lumen enlargement *in vivo*. These results redefine VM progression as a process driven by competitive cellular processes that could promote clonal expansion of the TIE2 mutant cells (44). Given that clonal expansion drives lesion growth in vascular anomalies such as cerebral cavernous malformations and AVMs (45–47), it is plausible that it similarly contribute to venous malformation progression.

While the PI3K/AKT and MAPK/ERK signaling pathways can drive endothelial cell proliferation and morphogenesis (48, 49), small GTPases (RHO, RAC, and CDC42) play a crucial role in endothelial cell migration, sprouting, vessel stability, polarity and lumen formation by regulating the actin cytoskeleton (50–57). A seminal study led by Dr. George Davis demonstrated that inhibition of Rho GTPases by Clostridium difficile toxin B can abrogate lumen formation in a 3D collagen model (58, 59). Subsequent studies refined the specific contributions of individual GTPases and determined that CDC42, RAC1, and their effectors PAK2 and PAK4, play a key role in lumen formation. Of note, a recent study by Aw and colleagues demonstrated that RAC1 signaling is dysregulated in PIK3CA-driven VM. In this study, inhibition of the RAC1/PAK1 axis rescued the vascular architecture and abnormal growth in a microphysiological *in vitro* model of VM, highlighting RAC1 as a potential therapeutic target in PIK3CA driven VMs (17). Consistent with the importance of RAC1, Cetinkaya and colleagues showed that a loss of function mutation in ELMO2 disrupts RAC1 activation and contributes to malformed blood vessels in intraosseous vascular malformation (60). Studies on CDC42 revealed that it is equally critical for vascular morphogenesis by promoting endothelial migration, filopodia formation and sprouting, while its deletion can lead to capillary venous malformation in the retina (42). Conversely, excessive CDC42 activity in brain ECs, in the context of Rbpj deficiency, led to disrupted cell migration and polarity, contributing to brain AVMs (61). On note, CDC42 deletion in mural cells can also result in impaired vascular integrity and dilated capillaries (62).

In summary, the dysregulation of the TIE2/PI3K/AKT pathway is central to the pathogenesis of VM, and several studies to date support its role in promoting endothelial cell hyperplasia, abnormal vessel morphogenesis and impaired vascular stability. Other signaling pathways have been implicated in VM, such as the MAPK signaling pathway, and small GTPases like RAC, RHO, and CDC42. Future research should focus on the latter molecular effectors to understand their role in the VM-genesis and determine the potential of these targets for effective therapies with single or dual agents.

## Diagnosis

Clinically, VMs typically present as soft, compressible, bluish lesions that enlarge with time (2). Chronic pain, swelling, localized thrombi, and bleeding are common complications (2). VM lesions are diagnosed through clinical assessment supported by imaging. Ultrasound is often the first line of modality, offering a non-invasive system to visualize vascular lesions (63–65). Doppler ultrasound helps differentiate between low-flow and high-flow vascular lesions (63, 66) as VM typically appears as hypoechoic, compressible lesions with slow or absent flow signals, in contrast AVMs displays pulsatile, and high-velocity signals. However, ultrasound ability to assess deep or extensive lesions can be limited. Magnetic Resonance Imaging (MRI) is considered the gold standard for the diagnosis for VMs, offering high resolution of lesion and its involvement with surrounding tissue. VM lesions typically appear hyperintense on T2-weighted images and hypointense to isointense on T1, often with multilocular cystic spaces and phleboliths; importantly, they show slow, delayed enhancement without early arterial inflow, differentiating them from AVMs or arteriovenous fistulas (67, 68). Lymphatic malformations, by comparison, appear as predominantly non-enhancing, fluid-filled cystic lesions without significant venous components.

In more complex cases, venography may be used to evaluate venous drainage patterns. While radiographs and CT scans are less commonly used in the diagnosis of VMs, they can be useful for detecting calcifications and deeper tissue involvement (69).

Combining clinical and imaging findings enables precise classification of VMs which is critical for differentiating them from other vascular anomalies and guiding appropriate treatment strategies. Although TIE2 and PIK3CA mutations are known to be present in VM, performing biopsies to check for these mutations is not a routine procedure due to the invasive nature and the cost for sequencing. Recent VASCERN-VASCA recommendations highlight the growing clinical utility of genetic testing for somatic variants in vascular malformations, emphasizing standardized gene panels, optimized variant calling, and careful interpretation to improve diagnostic accuracy and guide precision-based (70). Determining the causative mutation in patients would ease diagnosis and personalize the therapy to improve efficacy. Efforts should focus on developing cost-effective sequencing techniques with high sensitivity for low allelic frequency, that can be used for non-invasive fluid biopsies.

## Treatment

VM lesions can be extensive in size and multifocal, invade surrounding tissue and organs, and are often associated with life-threatening morbidities such as hemorrhage and coagulopathy. For these reasons, complete excision of VMs is often not possible, and treatment is primarily aimed at managing symptoms and improving the patients' quality of life.

To manage symptoms of pain and inflammation, NSAIDs such as aspirin and celecoxib are commonly prescribed (71–73). In some cases, anticoagulants like low-molecular weight heparin and direct oral anti-coagulants are used to alleviate pain and reduce localized intravascular coagulopathy (LIC) (4, 74–76).

Sclerotherapy remains the first line of treatment for many VMs, particularly when lesions are localized and accessible. The procedure involves the injection of a sclerosing agent (sodium tetradecyl sulfate, ethanol, or bleomycin) that promotes endothelial cell lysis and coagulation within the affected vein, leading to vessel closure. Surgical excision is considered when a VM is small, accessible, and patients do not present risk for bleeding or coagulopathy. It can be used in conjunction with sclerotherapy to remove residual tissue after lesion shrinkage (64, 77–79). However, these approaches are not always curative, and lesions often regrow.

Recent advances in the understanding of molecular mechanisms of VM have led to the development of therapies targeting the PI3K/mTOR signaling pathway. Sirolimus, a specific mTOR inhibitor, originally used in transplant recipients to prevent organ rejection, has shown efficacy in reducing VM lesion size and improved symptoms in several clinical trials (80–83). Systemic sirolimus can have some adverse effects leading to need for alternative delivery method. Recent clinical trials of topical sirolimus showed promising results with reduction in blebs, bleeding, and lesion size (84–86). Another agent under investigation is Alpelisib, an inhibitor of the PI3K pathway, which demonstrated significant reduction in VM size, offering hope for the future of clinical applications (16, 87). Other investigational drugs like Miransertib, a pan AKT inhibitor have been reported to improve mobility and quality of life while promoting lesion stabilization (88, 89). More recently, Rebastinib, a TIE2 and ABL inhibitor, has showed significantly reduction in VM lesion size, and visible lightening of lesion coloration with no adverse effects reported to date, making it promising for VM treatment (90). However, larger studies and long-term research are necessary to confirm these findings. Table 2 summarizes the clinical efficacy, and adverse events of targeted therapies. Ongoing investigations are critical for translation of these targeted therapies into safe and effective therapeutic options for patients with VMs.

**Table 2:** Summary of targeted therapies efficacy, dose response and adverse events.

Category	Therapy	Target	Outcome	Adverse events	References
FDA approved for of PIK3CA-Related Overgrowth Spectrum (PROS)	Alpelisib	PIK3CA inhibitor	Reduced pain, 20% reduction in lesion size, 60% responders have response lasting ≥12 months.	Hyperglycemia, diarrhea, stomatitis/mucositis, insulin resistance and growth resistance	(16, 87, 111, 112)
FDA approved for transplant but not for VM	Sirolimus	mTOR inhibitor	20%–40% reduction in lesion size, reduction in pain, response sustained for months to years on medication—disease progression occurs with dose reduction or discontinuation	Mucositis, pneumonitis, and upper respiratory infection	(40, 82, 83, 113, 114)
Clinical Trial	Topical Sirolimus	mTOR inhibitor	Reduction in blebs, bleeding, reduced recurrent bleeding, reduced lesion size,	Irritation at application site, nasopharyngitis and pyrexia	(84–86)
Clinical Trial (PROS/Vascular Anomalies)	Miransertib	Pan AKT inhibitor	Stabilized lesion, improved mobility, quality of life	Neutropenia, Metabolic effects (blood insulin elevation, hyperglycemia), Mucositis/stomatitis and rarely deep vein thrombosis	(88, 89)
Clinical trial (Cervicofacial venous malformation)	Rebastinib	cABL/TIE2 inhibitor	Reduced VM lesion size, lightening in color	No adverse effect reported	(90)

## Models of venous malformation

**Figure 2:**
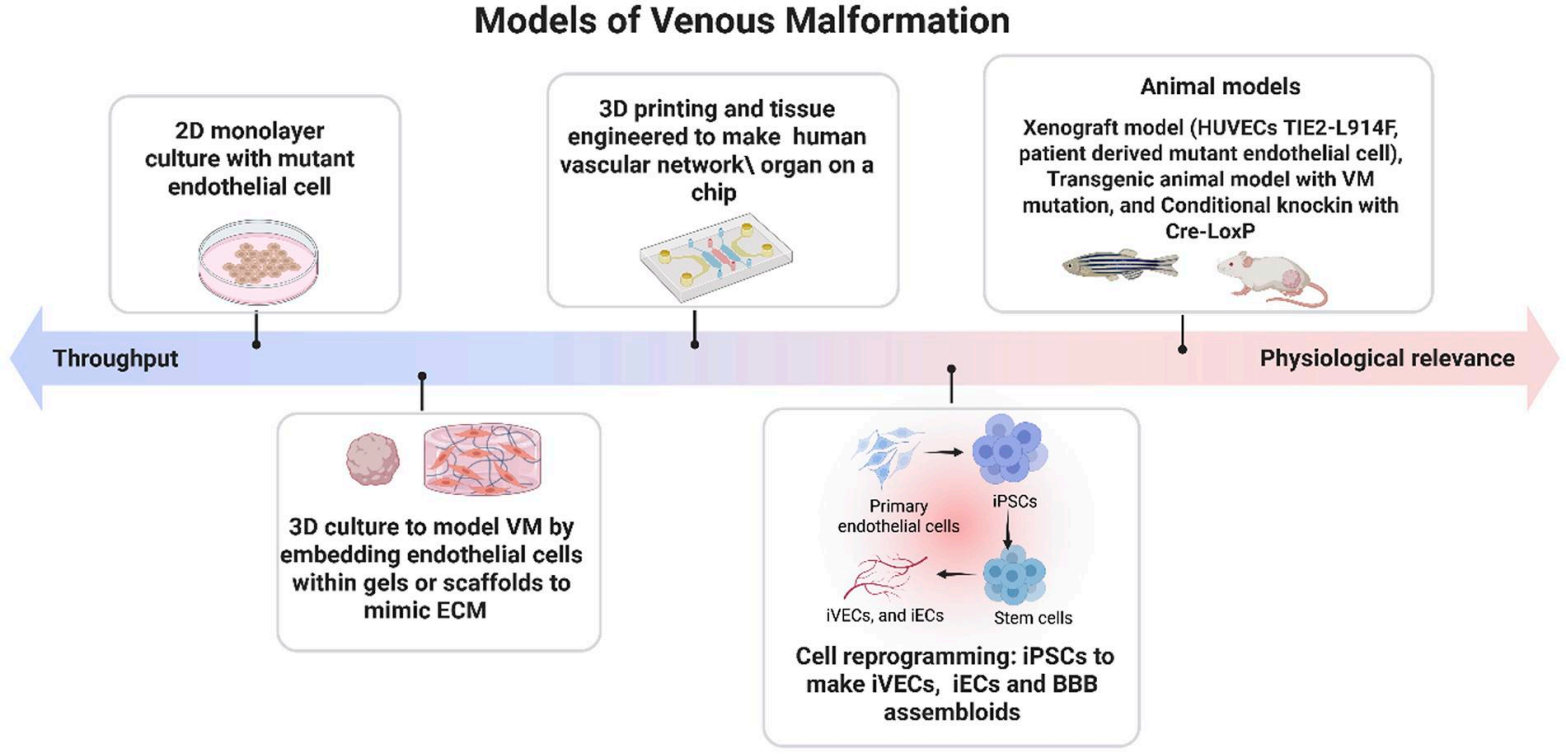
Experimental models of venous malformation. Experimental systems used to study venous malformations range from high throughput 2D cell culture systems to physiologically relevant 3D engineered tissues, xenograft models, genetically modified animals, and emerging humanized platforms. These models differ in complexity, throughput capacity, and their ability to recapitulate native vascular structure and signaling. Strategic integration of multiple model types—balancing experimental efficiency with biological fidelity—enables more accurate investigation of disease mechanisms and pathological progression in venous malformation. Created in BioRender. Sagar, K. (2026), licensed under Academic License.

Despite the advances in the treatment of VMs, substantial challenges remain in achieving complete and lasting resolutions. These challenges can be resolved by deeper understanding of the pathophysiology of VMs. To bridge this gap, researchers have developed experimental models designed to recapitulate VM biology. These include *in vitro*, 3D bioengineering platforms, and *in vivo* models, with each providing unique strengths and inherent limitations in their ability to mimic disease (Figure 2). In the following section, we will provide a detailed and critical analysis of these model systems.

### *In vitro* models

*In vitro* models are essential for investigating molecular and cellular mechanisms underlying VMs in a controlled environment. These can be classified into 2D and 3D cell cultures. 2D models are widely used due to their simplicity and ease of implementation. Typically, these models involve culturing endothelial cells in medium supplemented with growth factors. A common 2D model for VMs involves the use of endothelial cells, such as human umbilical vein endothelial cells (HUVEC) harboring VM causative mutations in TIE2 or PIK3CA. These cell lines are generated by retroviral transduction with a vector for expression of mutant *TIE2* or *PIK3CA*. This method consists of overexpression of the mutant gene, thereby it is of paramount importance to generate a control line overexpressing the wild-type (WT) *TIE2* or *PIK3CA.* This 2D model allowed for defining downstream pathways such as PI3K/AKT and MAPK, and mutant cell properties such as increased migration, cell survival and angiogenesis. A more informative 2D approach is based on the use of VM patient-derived EC with *TIE2* and *PIK3CA* mutations, as they uphold unique patient genetic background and do not rely on overexpression of the mutant allele (9, 18, 91). However, 2D systems lack spatial organization, tissue specific extracellular matrix and environmental cues such as blood flow.

3D culture models can offer a more accurate and physiologically relevant representation of the *in vivo* environment. These are powerful tools for studying VMs because they involve embedding endothelial cells within gels or scaffolds that closely resemble native extracellular matrix composition and architecture. This approach provides more realistic cell-cell, cell-microenvironment and dynamic behavior of cells. A widely used 3D *in vitro* model of lumen formation and angiogenesis was generated by embedding endothelial cells seeded onto cytodex beads into fibrin gel matrix, which served to simulate vascular ECM (92). Fibroblasts are added on the top of the gel, to support lumen formation by secreting angiogenic factors such as collagen I. In this system, sprouting is visible on day 2, lumen formation by day 4 and extensive branching by day 10. This approach has been successfully adapted to investigate the impact of VM-associated mutation on endothelial angiogenesis, providing insights and potential therapeutic target (41, 93). While normal HUVECs formed organized tube-like lumens, TIE2-mutant HUVECs developed dilated, saccular channels resembling the malformed vessels seen in VM lesions. In addition to fibroblasts, researchers have co-cultured smooth muscle cells and pericytes with endothelial cells, further enhancing the physiological relevance of the vascular architecture (41, 94).

Recently, Aw and colleagues developed a microfluidic device which incorporates a physiologically relevant ECM, and shear stress to mimic blood flow (17, 95). This device uses fibrin-based hydrogels as a matrix, wherein hydrogels are infused with Alexa-fluor conjugated fibrinogen for visualization of ECM degradation, allowing real time imaging of vascular lesion formation. When PIK3CA-E542K endothelial cells were embedded in the hydrogel-based microfluidic device, they formed dilated and irregular lumens within 4 days, similar to VM lesions in patients and showed impaired mechanotransduction under shear stress.

Another 3D *in vitro* angiogenic model was developed by Apeksha and colleagues in 2024, by integrating traction force microscopy with cellular invasion to study mechanical forces driving a venous-like malformation known as cerebral cavernous malformations (96). In this model, endothelial cells knocked-down for the CCM2 gene were co-cultured with wild-type endothelial cells. *CCM2*-deficient cells exerted elevated traction forces and remodeled ECM, guiding migration of WT cells, demonstrating that mechanical cues and cell-ECM interactions contribute to lumen formation and vascular remodeling. Collectively, these findings highlighted the ability of 3D *in vitro* platforms to recapitulate critical biomechanical and cell-matrix interaction events underlying venous malformations. However, despite their strengths, *in vitro* systems rely heavily on primary endothelial cells, which include inherent limitations including possible loss of venous identity over time, and lack of patient-specific genetic background.

To overcome these challenges, researchers are now increasingly using human induced pluripotent stem cells (iPSCs). Shortly after the Yamanaka factors' discovery (97), it became possible to differentiate iPSCs into arterial, venous, and lymphatic characteristic, providing a platform to use iPSCs to model vascular disease. The first major breakthrough in VM modelling came when Pan and colleagues developed iPSC-derived venous endothelial cells (iVECs) with patient-specific TIE2-L914F mutation (98). These iVEC recapitulated the hallmark phenotypes including VM-vessel dilation, and sparse smooth muscle cell coverage, both *in vitro* experiments and in *in vivo* xenografts. Importantly, this study emphasized that disease phenotype is specific to venous, rather than arterial, highlighting the necessity of precise lineage specification in modelling venous malformation. Furthermore, Lazovic and colleagues developed an iPSC-derived endothelial cell (iEC) carrying TIE2-L914F mutation at its endogenous locus using CRISPR-Cas9 (99). The TIE2-L914F iECs showed reduced TIE2 expression compared to TIE2-WT (wild-type), yet the pathway remained highly activated with increased phosphorylation of TIE2 and AKT. These cells exhibited altered angiogenic markers (PIK3R1, PIK3R2, ANG2, VEGFA, PDGFB, CXCL12, ITGA5/ITGB1, among others), enhanced migration, and reduced alignment in response to shear stress, while proliferation remained unaffected. In addition to mutant TIE2 model, hiPSCs carrying PIK3CA H1047R variant have also been used to study the effects of activating mutations on cellular behavior. Madsen and colleagues showed homozygous PIK3CA H1047R hiPSCs are more likely to support tumorigenesis as they maintain self-stemness through NODAL/TGFβ signaling, conversely heterozygous cells display minimal phenotypic alterations (100). This could explain why PIK3CA mutation in VM endothelial cells, which are always found in heterozygosis, do not fuel cellular transformation or tumorigenesis.

iPSC-derived vascular models can also be applied to generate organoids to mimic specialized structures such as the blood brain barrier (BBB). BBB assembloids were generated by fusing brain and vascular organoids from patient iPSCs (101) to model BBB disruption in CCM patients. The future use and improvement of organoid systems, particularly with the use of iPSCs, has a great potential to advance the research in VMs by providing physiologically relevant models.

Although advances *in vitro* platforms, ranging from primary endothelial culture to genome-edited iPSCs derived endothelial cells and vascular organoids, have transformed our ability to dissect the cellular and molecular drivers of VMs, none of these systems are sufficient to capture the full spectrum of the disease.

Despite the numerous advantages and flexibility of these systems, *in vivo* models still hold the best potential to integrate all together the genetics, cellular processes, heterotypic cell interaction and hemodynamic forces that shape lesion development and progression.

### *In vivo* models

The first murine model of VM was developed in 2009 using Tet-On conditional transgenic system, by controlling the expression of the murine polyomavirus middle T antigen (PyMT) gene (102). By microinjecting the PyMT into the fertilized mouse eggs, transgenic mice developed vascular lesions, histologically similar to VM. While this was a valuable method to study VM pathogenesis, it has not been used extensively, as the model failed to account for the genetic causes of VM, such as mutation in TIE2 and PIK3CA found in VM patients.

One of the first significant breakthroughs in this area came with the work of Boscolo and colleagues in 2015. They developed a mouse model to accurately mimic human VMs by injecting HUVECs expressing TIE2-L914F mutation in immune compromised mice (40). Mice injected with HUVEC TIE2-L914F developed VM-like lesions characterized by ectatic, slow flow, blood filled channels with aberrant perivascular cell coverage. Lesions progressively enlarged with time, mimicking the progressive nature of VM. In subsequent studies, the same team of investigators refined the understanding of VM by isolating and characterizing ECs from both lesional tissue and lesional blood samples from VM patients (18, 103, 104). These patient-derived VM-ECs injected into nude mice also formed large blood-filled vessels with scarce smooth muscle cell coverage. While xenograft models have been valuable tools in drug discovery and early-stage testing, they do not fully recapitulate events happening during embryonic development, and they lack the immunological and microvascular environment. These limitations highlight the need for genetically engineered mouse models.

Firstly, Castel and colleagues generated a transgenic mouse model with PIK3CA H1047R mutation using Cre-loxP method with Sprr2f-Cre strain specific to uterine epithelial cells and TIE2-cre specific to endothelial cells, which led to development of spinal and skin vascular lesions with classical VM phenotype like vascular dilation, hemorrhage, and cavernous spaces (11). Skin lesions were found in 90% of mice, showing features of human VM, including CD31+staining, and hemosiderin deposition, while negative for markers of other vascular malformations such as infantile hemangioma (GLUT-1, and WT-1) and lymphatic malformation (LYVE-1, PROX-1). A concomitant study by Castillo and colleagues developed a mouse model with mosaic expression of endogenous PIK3CA H1047R in embryonic mesoderm (10). Mosaic expression of PIK3CA H1047R was induced by crossing PIK3CA WT/H1047R mice with T-CreERT2 mice which driven by mesoderm specific T (Brachyury) promoter. Low dose tamoxifen administration at E7.5 enabled heterozygous and mosaic expression of human PIK3CA. This model showed significant cell hyperproliferation, reduced pericyte coverage and loss of arteriovenous differentiation. These studies provided the first direct connection between hyperactive mutant PIK3CA and VMs and reported preclinical efficacy of the mTOR inhibitor rapamycin and the PIK3CA inhibitor Alpelisib.

The first groundbreaking report of inherited *TIE2* mutations in VMs was published by Vikkula and colleagues back in 1996 (34). However, murine models carrying *TIE2* mutations have not been available until 2025 when a study by Bischoff and colleagues reported the generation of a transgenic mouse line with conditional expression of the *TIE2-L914F* allele from the endogenous *Rosa26* locus (105). This mouse was crossed with a constitutive endothelial-specific *Tie2-Cre* driver to investigate the effects of the TIE2 mutation during early vascular development. Mutant embryos consistently died around E9.5 exhibiting malformations of the blood vessels and severe remodeling defects in the vasculature of the yolk sac. This work provides the first functional evidence that the somatic TIE2-L914F mutation cannot be tolerated during vascular development, explaining why this mutation is never inherited. Future studies on the late-embryo and postnatal mosaic expression of TIE2-L914F in the vasculature have the potential to enhance our understanding of the cellular processes and molecular mechanisms involved in VM lesion formation. Such a model would be a great tool for preclinical studies and to investigate the mechanisms of action of current therapeutical approaches.

In addition to *in vivo* transgenic mouse models, zebrafish are also a powerful model to study vascular development and to dissect the genetics of venous malformations. Expression of the TIE2-R849W in zebrafish via microinjection at one cell stage resulted in malformations of the caudal vein plexus and craniofacial vascular defects (106). Supporting these findings and the suitability of zebrafish to model vascular malformations, Bell and colleagues characterized TEK and MAP2K1 variants of uncertain significance in zebrafish, revealing that the TEK mutation generated VMs, whereas the MAP2K1 mutations triggered AVMs (107). They also demonstrated that compound TEK mutations in cis (i.e., on the same allele) have additive effects, leading to a more severe disease phenotype. Wang and colleagues identified ACTA2 as a candidate VM driver gene through proteomics and high connotation functional gene screening (HCS) (108). Using zebrafish, ACTA2 silencing by morpholino antisense oligonucleotide resulted in defective vascular formation, compromised vessel integrity, and microvascular malformations, effectively recapitulating features of human VMs.

Collectively, these findings underscore the pathogenic contribution of these genetic mutations but also highlight the potential of these models as preclinical platforms to test the therapeutic efficacy of candidate targets in VMs.

## Summary and future directions

Recent advances have significantly improved our understanding of VM biology, highlighting the central role of genetic mutations such as TIE2 and PIK3CA. Despite this genetic knowledge, venous malformations remain challenging to treat effectively, and FDA approved therapies are lacking. Current therapies in clinical trial, such as Sirolimus and Alpelisib, fail to provide complete and lasting resolutions, highlighting the need for more precise approaches. *In vitro* systems-including 2D and 3D culture, microfluidic devices, and patient derived iPSC models have provided mechanistic insight into vascular remodeling, while *in vivo* models, ranging from xenograft to genetically engineered mice to zebrafish, have allowed functional validation of candidate genes and preclinical testing of therapies.

Looking forward, emerging technologies such as 3D bioprinting and organ-on-chip platforms present exciting opportunities to model VMs with high physiological reliability. Perfusable, bio printed microvascular networks could be seeded with patient specific mutant endothelial cells to recapitulate human VM architecture, microenvironment, and hemodynamic forces. Integrating these approaches with existing *in vitro* and *in vivo* models will enable precise, patient tailored investigation of disease mechanisms and therapeutics, bridging gap between molecular discovery and clinical translation. Collectively, these advances promise to accelerate the development of personalized therapies and improve outcomes of patients with venous malformations.
